# Phonetic-acoustic and ultrasonographic characteristics of speech after lingual frenectomy: a case report

**DOI:** 10.1590/2317-1782/e20240202en

**Published:** 2025-08-08

**Authors:** Águida Alves Pereira, Danielle Pereira de Lima, Aline Natallia Simões de Almeida, Pablo Vinícius do Nascimento Pinto, Rômulo César de Alencar, Ithalo José Alves da Silva Cruz, Nyedja Tatyane Pereira Alves, Zulina Zouza de Lira, Daniele Andrade da Cunha, Hilton Justino da Silva

**Affiliations:** 1 Departamento de Fonoaudiologia, Universidade Federal de Pernambuco – UFPE - Recife (PE), Brasil.; 2 Departamento de Odontologia, Universidade Federal de Pernambuco – UFPE - Recife (PE), Brasil.

**Keywords:** Ankyloglossia, Speech Acoustics, Speech Therapy, Lingual Frenulum, Clinical Case

## Abstract

This case report aimed to verify the effect of lingual frenectomy on the functional anatomical aspects of the tongue, the phonetic-acoustic characteristics, and the magnitude of tongue movement in the phonemes [ɾ] and [l] after the lingual frenectomy. The anatomical characteristics of the lingual frenum and the functional aspects of the tongue were evaluated using the Protocol for Evaluation of the Lingual Frenum. The phonetic-acoustic particularities of speech were assessed through formant analysis using PRAAT software, and the evaluation of the magnitude of tongue movements was conducted via ultrasonographic analysis with Articulate Assistant Advanced (AAA) software. After the assessments, the patient was referred for the lingual frenectomy and was reevaluated after 7 and 14 days of healing. It was observed through the functional anatomical evaluation that the patient showed modifications in the shape of the tongue tip, greater elevation of the tongue in the oral cavity, and improvement in the contact of the tongue tip with the labial commissures. The acoustic evaluation of speech and the ultrasonographic assessment of tongue movements indicated a longer emission time for the words, increased verticalization and anteriorization of the tongue during speech production, which were more evident for the phoneme [ɾ]. Thus, the instrumental evaluations contributed to the clinical assessment, facilitating the observation of the patient’s progress after the lingual frenectomy, identified in the analysis of the formants and highlighted through the ultrasonographic analysis of the tongue

## INTRODUCTION

Speech is a motor act that depends directly on the balance between the anatomical and functional structures of the stomatognathic system. Articulating sounds involves the coordination of rapid, synchronous, and precise movements of the tongue, lips, and jaw. Changes in this balance can interfere with the proper production of speech sounds, especially when the sounds produced depend on the movement of the tip of the tongue^(1-3)^.

Brazilian Portuguese (BP) consonants are classified according to three parameters: articulatory mode, place of articulation, and voicing. Based on the articulatory mode, consonants can be classified into different categories, including laterals and taps. The [l] and [ʎ] sounds are classified as lateral regarding the articulation mode and dental or alveolar regarding the articulation point. The [ɾ] sound is characterized as a tap regarding the articulation mode. Taps are articulated using a single, rapid, punctual movement, briefly obstructing the passage of air in the alveolar region. That is, the obstruction results from the contact between the tip of the tongue and the alveoli. Thus, two articulating points (the tip of the tongue and alveoli) meet in the [l], [ʎ], and [ɾ] sounds^(4)^. Thus, considering the need for free movement of the tip of the tongue to articulate these sounds, children with abnormal lingual frenulum tend to have speech changes^(5)^.

Lingual frenulum disorder, commonly known as “tongue tie,” is defined as a congenital oral anomaly called ankyloglossia. It is characterized by a small portion of tissue on the sublingual surface that restricts the movement of the tongue^(1-6)^. Ankyloglossia can lead to different functional limitations, such as difficulties in breastfeeding, chewing, limitations in cleaning the oral cavity, difficulty swallowing, changes in breathing, speech difficulties, and psychosocial stress^(7,8)^.

The surgical procedure indicated in cases of abnormal lingual frenulum is lingual frenectomy, which should be recommended after careful evaluation of the morphological and functional aspects of the tongue^(9)^. The range of tongue movements is expected to improve after the procedure, possibly having a positive impact on the production of the [ɾ] and [l] phones^(9)^.

However, speech-language-hearing assessment and monitoring is essential before, during, and after lingual frenectomy, since distortions in speech sounds and functional difficulties may persist even after anatomical release.

Anatomical and functional assessment, associated with complementary ones, can help identify the impact of lingual frenectomy on these aspects. The acoustic analysis of speech allows the identification of specific sound characteristics, such as formants, which are acoustic events that can be systematically associated with the position of the tongue during sound production^(10,11)^.

Ultrasound (US), in turn, allows the evaluation of tongue movements by placing a transducer in the submandibular region, generating images that demonstrate the magnitude of lingual movements during the articulation of sounds and the coordination of rapid and synchronous tongue movements^(12,13)^.

Using instruments such as US and acoustic analysis of speech in patients with ankyloglossia can enrich the evaluation and therapeutic monitoring in this area. This case report analyzed in detail the relationship between the anatomical and functional aspects of the tongue and these evaluations, helping to understand tongue mobility during speech production. These instruments can also be incorporated into the professional’s clinical routine in evaluation and therapeutic monitoring with biofeedback in patients with ankyloglossia.

Thus, this research aimed to verify the effect of lingual frenectomy on the anatomical and functional aspects of the tongue, phonetic-acoustic characteristics, and the magnitude of tongue movement in the [ɾ] and [l] phones after lingual frenectomy.

## CLINICAL CASE PRESENTATION

This research was approved by the institution's Ethics and Research Committee under approval number 6,588,482. The study participant’s legal guardians received and signed an informed consent form. This case study followed the recommendations of the international CARE guideline for publications of case reports^(14)^.

A 10-year-old female patient with an abnormal lingual frenulum participated in this study. She was submitted to the Lingual Frenulum Assessment Protocol^(15)^.

The main complaint in the medical history survey referred to speech, especially the production of the [ɾ] voiced alveolar tap phone. There was no family history of lingual frenulum changes, and the child had no health, respiratory, masticatory, or swallowing problems. She had no vocal, auditory, or phonological changes or any history of previous lingual frenectomy. Regarding sucking, the mother reported breastfeeding without discomfort for more than a year, when weaning began.

The evaluations performed on the patient will be described below. All of them were conducted at three moments: before lingual frenectomy (T1), 7 days after frenectomy (T2), and 14 days after frenectomy (T3). Chart 1 presents a chart of the study’s evaluation moments and the procedures performed in each one.

**Chart 1:** Assessment moments and procedures

Before lingual frenectomy (T1)		After 7 days of lingual frenectomy (T2)	After 14 days of lingual frenectomy (T3)
Procedures		Procedures	Procedures
Medical history survey	LINGUAL FRENECTOMY	Functional anatomical assessment	Functional anatomical assessment
		Acoustic assessment	Acoustic assessment
Functional anatomical assessment		Ultrasound assessment	Ultrasound assessment
Acoustic assessment			
Ultrasound assessment			

### Functional anatomical evaluation of the tongue

The clinical examination began by measuring the maximum mouth opening and maximum mouth opening with the tip of the tongue touching the incisive papilla, using a digital caliper manufactured by the Electronic Digital Caliper.

Next, the lingual frenulum was evaluated by observation, starting with tongue elevation. The shape of the tip of the tongue, fixation of the lingual frenulum on the floor of the mouth and the underside of the tongue, and the length and thickness of the lingual frenulum were observed.

A functional assessment was also performed based on the following observations: tongue mobility; tongue position at rest and during speech; speech assessment considering aspects of omission, substitution, and/or distortion; mouth opening during speech; lip participation; mandibular movement; and speech speed and accuracy.

The speaking test collected samples of informal speech, automatic speech, and picture naming, using the protocol's own boards.

### Acoustic assessment of speech

The acoustic collection of the child's speech was performed using the PRAAT software, version 6.4.05^(16)^. The spectrogram settings of the software were adjusted to generate wide-band spectrograms. Spectrogram settings were selected in the spectrum menu, and the window length was changed to 0.0043 in the wide-band option to extract the formants of the target phones.

The patient was seated in an armchair, with her torso erect and her gaze directed toward the horizon. A Karsect HT-9 microphone was positioned approximately 3 cm from the oral cavity. The microphone was connected to an Andrea PureAudio USB adapter, which was connected to the evaluator's computer that ran the PRAAT software.

The patient was instructed to emit two carrier sentences: one containing the [ɾ] and the other the [l] target phones. The sentences were created especially for this study to generate speech samples in a similar phonetic context, thus extracting the formants of the [ɾ] and [l] phones in the stressed syllables of the Portuguese words *“parada”* and *“palada”*.

The sentences were, “Say *‘parada’* quietly” and “Say *‘palada’* quietly”. The emissions were recorded in the software for later analysis and extraction of formant measures, as well as the duration of word emissions.

The words “say” and “quietly” were removed from both carrier sentences for acoustic analysis in PRAAT, as their function was only to provide a similar phonetic context. The duration of the words *“parada”* and *“palada”* was extracted from these initial excerpts.

Then, the [ɾa] and [la] stressed syllables were extracted from the words, and next the vowels were removed, leaving only the [ɾ] and [l] target phones. With these segments isolated in the spectrogram, the cursor was positioned in the center of the emission, and the formants F1, F2, and F3 were extracted.

### US assessment of tongue movements

The US assessment used the Articulate Assistant Advanced (AAA) software, version 217.02. It was installed on a Dell computer and coupled to an ultrasound transducer. The patient was seated in an armchair, with her torso forming a 90° angle and her gaze directed toward the horizon.

Specific carrier sentences were used to capture tongue movements during [ɾ] and [l] phone production. We chose a sentence in which the target phones were inserted between sounds that were easy to analyze in the US image. Thus, the vowel [a] was chosen due to its articulatory parameters – it is a low vowel concerning the height of the tongue in the vertical dimension, central concerning the anteroposterior displacement of the tongue, open concerning the movement of the jaw, unrounded concerning the positioning of the lips, and oral concerning the closing movement of the soft palate^(4)^.

She was given the following commands “Repeat: ‘*diga ara, diga ara, diga ara’*” (say *“ara”*) and “Repeat: *‘diga ala, diga ala, diga ala’*” (say *“ala”*). The US images were recorded for later extraction of the magnitude of tongue movement in the anterior, middle, and posterior portions during phone production. These measurements were obtained by tracing the surface of the tongue with a spline.

**Figure 1:**
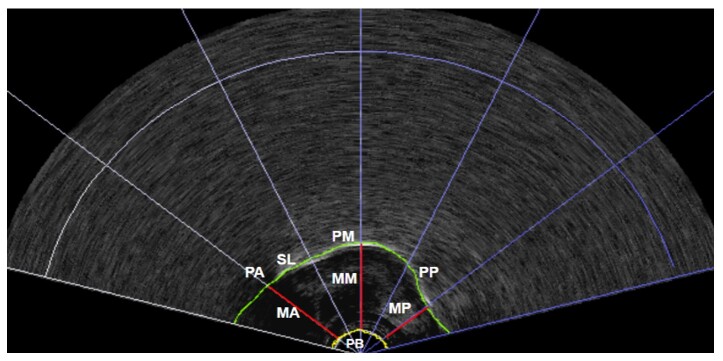
Ultrasound image with an example of the extraction of the anterior, middle, and posterior measures of the tongue

The US analyses of the tongue collected the distances between the base point of the transducer and the anterior, middle, and posterior portions of the tongue in the lowest position (start of the target phone articulation) and in the highest position (complete target phone articulation). This procedure was performed for both phones ([ɾ] and [l]) in the three evaluation moments: T1, T2, and T3 (Figure 1).

**Caption:** PA = anterior tongue; PM = middle tongue; PP = posterior tongue; SL = tongue surface; MA = anterior tongue measurement; MM = middle tongue measurement; MP = posterior tongue measurement; PB = base point. Source: The authors, 2024

### Lingual frenectomy

After completing all evaluations, the patient was referred for lingual frenectomy, performed by a dentist with experience in the area and a member of the research group. The patient returned for reevaluation after 7 (T2) and 14 days (T3) of healing.

The researchers performed speech-language-hearing photographic documentation, anatomical and functional reassessment of the tongue, acoustic assessment, and US assessment to enable subsequent comparisons and analyses.

## RESULTS

### Functional anatomical evaluation of the tongue

The patient obtained 5 points in T1’s clinical examination with the Lingual Frenulum Assessment Protocol – the best result is 0, the worst is 8, and 3 is the cutoff for an abnormal lingual frenulum. In tongue elevation, the tip was rectangular. The frenulum was attached to the floor of the mouth at the lower alveolar crest and to the lower surface of the tongue between the middle third and the tip. Moreover, the lingual frenulum was classified as thin.

**Figure 2:**
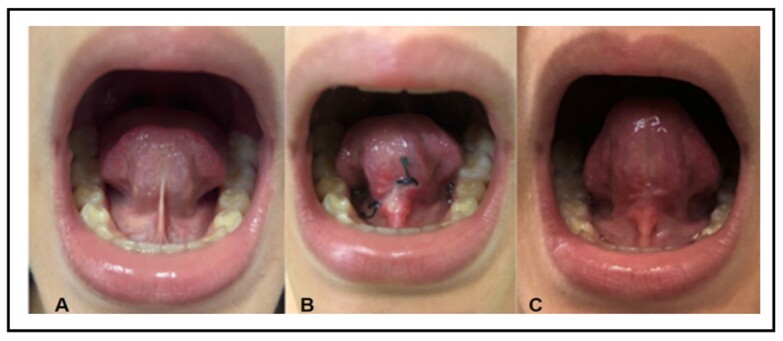
Tongue elevation before and after lingual frenectomy

The tongue elevation in the oral cavity increased after lingual frenectomy (at T2 and T3), with changes in its shape, which were more evident at T3 (Figure 2).

**Caption:** A = tongue elevation before lingual frenectomy; B = tongue elevation 7 days after lingual frenectomy; C = tongue elevation 14 days after lingual frenectomy. Source: The authors, 2024

The evaluation of mouth opening with the caliper found a progressive increase in millimeters (mm) between the evaluation times. In T1, the maximum mouth opening with the tip of the tongue touching the incisive papilla was 9 mm; in T2, 9.3 mm; and in T3, 10 mm. The relationship between maximum mouth opening and maximum mouth opening with the tip of the tongue touching the incisive papilla was 27.2% in T1, increasing to 30.3% in T3.

Sha also obtained 24 points in the functional tests at T1, on a scale whose best result is 0, the worst is 41; 20 points indicate possible interference of the lingual frenulum in tongue movement. The patient performed incompletely or not at all the following mobility test tasks: sucking the tongue on the palate, vibrating the tip of the tongue, protracting, touching the upper lip with the tip of the tongue, and touching the corners of the mouth with the tip of the tongue.

In protrusion, the tip of the tongue had a downward curvature pattern. When touching the upper lip with the tip of the tongue, a marked lip closure was observed. When trying to touch the corners of the mouth, asymmetry between the sides and a tendency for the tip of the tongue to turn downward were noted. It was impossible to observe the tongue position at rest because the patient had good lip closure.

**Figure 3:**
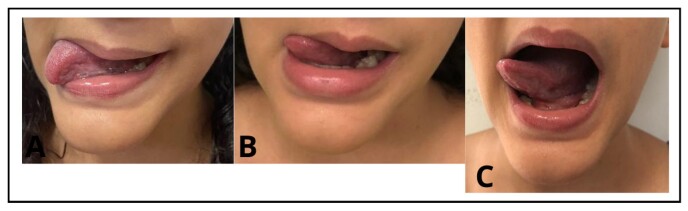
Touching the right corner of the mouth with the tip of the tongue

The functional assessments mentioned above were repeated at T2 and T3. Again, she performed the tasks incompletely or not at all, as before the surgery. However, she had greater amplitude and dexterity in touching the corners of the mouth (Figure 3).

**Caption:** A = Touching the right corner of the mouth with the tip of the tongue before lingual frenectomy; B = Touching the right corner of the mouth with the tip of the tongue 7 days after lingual frenectomy; C = Touching the right corner of the mouth with the tip of the tongue 14 days after lingual frenectomy. Source: The authors, 2024

Other aspects observed in the patient's speech at T1 included reduced mouth opening, tongue position on the floor both at rest and during speech, and imprecise articulation. In the reevaluations (T2 and T3), changes in the mandibular dynamics during speech were observed, more evident at T3.

In the speech test at T1, the patient obtained 9 points, with 0 being the best result and 12 being the worst. Distortion of the [ɾ] phone was observed in all speech samples. No phonetic distortion was observed in the [l] phone, indicating good articulation of this sound. The phonetic distortion of the phoneme [ɾ] remained at T3, despite the lingual frenectomy.

### Acoustic assessment of speech

The acoustic evaluation identified a longer emission of carrier words than in the initial evaluation and the reevaluations. Table 1 presents the duration in milliseconds (ms) of the words before and after lingual frenectomy.

**Table 1:** Length of emission of the carrier words *“parada”* and *“palata”* before and after lingual frenectomy

LENGTH OF THE TARGET WORD	[PARADA]	[PALADA]
T1	0.42 seconds	0.37 seconds
T2	0.43 seconds	0.57 seconds
T3	0.69 seconds	0.63 seconds

**Caption:** T1 = evaluation before frenectomy; T2 = evaluation 7 days after frenectomy; T3 = evaluation 14 days after frenectomy.

Source: The authors, 2024

Table 2 presents the values, in hertz (Hz), of the formants for the [ɾ] and [l] phones before and after lingual frenectomy.

**Table 2:** Formant measures before and after lingual frenectomy

FORMANTS	[ɾ]			[l] phone in Hz		
	T1	T2	T3	T1	T2	T3
F1	791.6	754.6	807.6	552.9	507.8	587.1
F2	2032.1	2198.9	2324.8	958.1	1386.2	1045.2
F3	3003.7	3030.5	2965.2	2219.7	1535.6	1296.8

**Caption:** T1 = assessment before frenectomy; T2 = assessment 7 days after frenectomy; T3 = assessment 14 days after frenectomy. Source: The authors, 2024

Changes in formant measures were observed at T2 and T3. The F1 and F2 values increased at T3 for both phones. Moreover, formant measures varied between T2 and T3. F3 decreased for both [ɾ] and [l], which was more pronounced for [l].

Changes were also found in the formant measures related to the transition of the target phones to the vowel [a]. F1, F2, and F3 increased for both phones when comparing the initial evaluation with the post-lingual frenectomy assessments.

### US evaluation of tongue movements in speech

The measures extracted from the anterior, middle, and posterior portions of the tongue changed between T1, T2, and T3. The measures increased in all portions of the tongue for the [ɾ] phone at all evaluation moments (Table 3).

**Table 3:** Ultrasound measures of the tongue in its low and high positions during the emission of target sounds

	LOW TONGUE					
	[ɾ]			[l]		
	T1	T2	T3	T1	T2	T3
Anterior portion	1.35	1.39	1.60^*^	1.58	1.64	1.63*
Middle portion	1.89	1.91	1.98*	1.91	2.04	1.64
Posterior portion	1.58	1.99	1.70*	1.78	2.40	1.75*
	HIGH TONGUE					
	[ɾ]			[l]		
	T1	T2	T3	T1	T2	T3
Anterior portion	1.72	1.70	1.78*	1.98	1.81	1.90
Middle portion	1.75	2.19	2.18*	1.96	2.36	2.04
Posterior portion	1.11	2.92	1.70*	1.92	2.35	1.48

**Caption:** T1 = evaluation before frenectomy; T2 = evaluation 7 days after frenectomy; T3 = evaluation 14 days after frenectomy

* Ultrasound measures that increased after reassessments

Source: The authors, 2024

As for the [l] phone, the measures were not systematic. The comparison between T1 and T2 found an increase in the elevation of the anterior, middle, and posterior portions of the tongue in the low tongue position, and an increase in the elevation of the middle and posterior portions in the high tongue position. These findings indicate a higher tongue posture in T2, which, however, was not maintained in all regions in T3 (Table 3).

Furthermore, the difference between the low and high tongue positions for [ɾ] phone production in the anterior and posterior portions of the tongue decreased at T3. For the [l] phone, this reduction was observed only in the anterior portion of the tongue (Table 3).

## DISCUSSION

This case report described the follow-up of a 10-year-old patient with lingual frenulum changes, who underwent lingual frenectomy, acoustic evaluation of speech, and US evaluation of tongue movements during speech.

The study observed clinical changes, such as greater elevation of the tongue within the oral cavity, change in the shape of the tip of the tongue, increased mouth opening with the tip of the tongue touching the incisive papilla, and better performance in touching the corners of the mouth with the tip of the tongue. These corroborate previous findings that observed improvement in the three-dimensional movement of the tongue in the oral cavity after 7 days of lingual frenectomy^(17)^. It was likewise found that successful lingual frenectomy improves the patient's tongue movement noticeably and immediately^(18)^.

The longer emission time of the words *“parada”* and *“palata”* in the acoustic analysis of speech can be attributed to the probable greater range of movement of the tongue and greater precision regarding the articulatory point. This finding agrees with the literature since ankyloglossia tends to restrict the tongue movement, leading to the omission or distortion of speech sounds and resulting in imprecise articulation^(3)^.

Regarding the evaluation of the formants of the [ɾ] and [l] phones, the increase in F1 and F2 values between T1 and T3 may be associated with greater mandibular opening, tongue verticalization, and tongue anteriorization. These findings agree with the literature, which indicates that the higher the F1 value, the greater the mandible vertical displacement; also, the higher the F2 value, the more anteriorized the tongue. The decrease in F3 value is associated with the increased size of the cavities due to the tongue position^(10)^. Therefore, it is understood that the lingual frenectomy positively changed the position of the tongue, detected acoustically.

This study found that the acoustic analysis of word duration played a relevant role in detecting changes in phone articulation during speech. The formant analysis presented consistent results for F1 and F2, indicating that it is a useful tool to assist in the evaluation and therapeutic monitoring of the patient's speech.

US analysis of the tongue identified greater elevation during the production of the [ɾ] phone, indicating a positive impact of lingual frenectomy on the amplitude of lingual movements in speech, corroborating the acoustic findings. Regarding [l], the inconsistent results can be attributed to the tendency for greater elevation of the tongue after the surgical procedure. However, this pattern was not maintained, since this phone did not present significant changes in speech, which led to an immediate increase in amplitude, followed by a return to the usual production pattern.

The reduced difference between low and high tongue position measures indicates a higher tongue position when producing the [ɾ] and [l] phones. The measures of the anterior and posterior portions of the tongue increased in [ɾ] articulation. This suggests an elevation of the tip of the tongue during speech, which may favor the rehabilitation of this phone. Regarding the posterior portion of the tongue, this finding may indicate possible articulatory compensations, more evident in individuals with ankyloglossia, as found in previous studies^(19)^.

It is important to emphasize that, despite the immediate gains after lingual frenectomy, the distortion in [ɾ] phone articulation persisted. Therefore, speech-language-hearing monitoring is essential to promote myofunctional balance, facilitating the adaptation of the patient's speech. It is recommended that this monitoring begins after surgical healing, which occurs in approximately 14 days, since it is known that anatomical release alone does not ensure functional rehabilitation, and other compensations may arise in the absence of speech-language-hearing intervention^(19)^.

The main limitation of this study lies in the acoustic analysis of formants and their association with tongue and jaw movement during speech production. Although the acoustic association with vowel production is well established in the literature, this relationship is not yet clearly defined for the liquid sounds of Brazilian Portuguese. However, despite the absence of such consolidated evidence, this case report found that the acoustic findings of formants indicated greater tongue movement in the vertical and anteroposterior axes after lingual frenectomy. Furthermore, the US identified these movements through images and measures, corroborating the clinical speech-language-hearing assessment. Thus, acoustic analysis and US are relevant instruments for the clinical practice of speech-language-hearing pathologists.

## FINAL COMMENTS

This case report of a 10-year-old child diagnosed with ankyloglossia and submitted to lingual frenectomy observed that the acoustic evaluation of speech and the US evaluation of the tongue can be incorporated into clinical speech-language-hearing practice to complement the clinical evaluation and the therapeutic process. These instruments provide objective measures of the tongue movement in the oral cavity and demonstrate potential to monitor the patient’s clinical evolution, also serving as a biofeedback resource during speech-language-hearing intervention.
