# Supplementary material for: Phonetic-acoustic and ultrasonographic characteristics of speech after lingual frenectomy: a case report
Source: Codas. 2025 Aug 8;37(4):e20240202. doi: 10.1590/2317-1782/e20240202en (PMC12337713; doi:10.1590/2317-1782/e20240202en)
Supplement: Translation

# Translation: Características fonético-acústicas e ultrassonográficas da fala pós frenectomia lingual: relato de caso

**DOI:** 10.1590/2317-1782/e20240202pt

**Authors:** Águida Alves Pereira, Danielle Pereira de Lima, Aline Natallia Simões de Almeida, Pablo Vinícius do Nascimento Pinto, Rômulo César de Alencar, Ithalo José Alves da Silva Cruz, Nyedja Tatyane Pereira Alves, Zulina Zouza de Lira, Daniele Andrade da Cunha, Hilton Justino da Silva

**Affiliations:** 1 Departamento de Fonoaudiologia, Universidade Federal de Pernambuco – UFPE - Recife (PE), Brasil.; 2 Departamento de Odontologia, Universidade Federal de Pernambuco – UFPE - Recife (PE), Brasil.

**Keywords:** Anquiloglossia, Acústica da Fala, Fonoaudiologia, Frênulo Lingual, Caso Clínico

## Abstract

Este relato de caso teve como objetivo verificar o efeito da frenectomia lingual nos aspectos anatomofuncionais da língua, nas características fonético-acústicas e na magnitude de movimentação de língua nos fones [ɾ] e [l] pós frenectomia lingual. As características anatômicas do frênulo lingual e funcionais da língua foram avaliadas com o Protocolo para Avaliação do Frênulo Lingual. As particularidades fonético acústicas da fala foram avaliadas por meio da análise dos formantes no software PRAAT e a avaliação da magnitude dos movimentos da língua foi realizada por meio da análise ultrassonográfica com o software Articulate Assistant Advanced (AAA). Após as avaliações a paciente foi encaminhada para realização da frenectomia lingual e foi reavaliada após 7 e 14 dias de cicatrização. Foi observado, através da avaliação anatomofuncional que a paciente apresentou modificações no formato do ápice da língua, maior elevação da língua na cavidade oral e melhora do toque do ápice de língua nas comissuras labiais. Com a avaliação acústica da fala e ultrassonográfica dos movimentos da língua foi verificado maior tempo de emissão dos vocábulos, maior verticalização e anteriorização de língua durante a produção da fala, as quais foram mais evidentes para o fone [ɾ]. Assim, as avaliações instrumentais contribuíram com a avaliação clínica, favorecendo a observação das evoluções da paciente após a frenectomia lingual, as quais foram identificadas na análise dos formantes e ressaltadas a partir da análise ultrassonográfica da língua.

## INTRODUÇÃO

A fala é um ato motor que depende diretamente do equilíbrio entre estruturas anatômicas e funcionais do sistema estomatognático. Articular o som envolve a coordenação entre movimentos rápidos, sincrônicos e precisos da língua, lábios e mandíbula. Alterações nesse equilíbrio podem interferir na produção adequada dos sons da fala, especialmente quando os sons produzidos dependem da movimentação do ápice da língua^(1-3)^.

As consoantes do Português Brasileiro (PB) são classificadas de acordo com três parâmetros: modo articulatório, ponto de articulação e vozeamento. A partir do modo articulatório, as consoantes podem ser classificadas em diferentes categorias, entre elas, as laterais e tepes. Os sons [l] e [ʎ] são classificados como laterais quanto ao modo e dental ou alveolar quanto ao ponto articulatório. Já o som [ɾ] quanto ao modo de articulação, é caracterizado como tepe. O tepe é articulado por meio de um único movimento, rápido e pontual, que provoca uma breve obstrução da passagem do ar na região alveolar. Isto é, a obstrução decorre do contato entre a ponta da língua e os alvéolos. Assim, nos sons [l], [ʎ] e [ɾ], observa-se o encontro entre dois pontos articuladores: ponta da língua e alvéolos^(4)^. Portanto, considerando a necessidade da livre movimentação da ponta da língua para articular esses sons, crianças com alteração de frênulo lingual, tendem a apresentar alterações na fala^(5)^.

A alteração do frênulo lingual, comumente conhecida como “língua presa”, é definida como uma anomalia oral congênita chamada anquiloglossia. É caracterizada por uma pequena porção de tecido na superfície sublingual que restringe a movimentação da língua^(1-6)^. A anquiloglossia pode acarretar diferentes limitações funcionais, como dificuldades na amamentação, mastigação, limitações na limpeza da cavidade oral, dificuldade de deglutição, alterações na respiração, dificuldades de fala e estresse psicossocial^(7,8)^.

O procedimento cirúrgico indicado em casos de alteração do frênulo lingual é a frenectomia lingual, que deve ser recomendado após cuidadosa avaliação dos aspectos morfológicos e funcionais da língua^(9)^. Após o procedimento, espera-se melhora na amplitude dos movimentos de língua e um possível impacto positivo na produção dos fones [ɾ] e [l]^(9)^.

Entretanto, é imprescindível a avaliação e acompanhamento fonoaudiológico antes, durante e após a frenectomia lingual, uma vez que distorções nos sons da fala e dificuldades funcionais podem persistir mesmo após a liberação anatômica.

A avaliação anatomofuncional, associada a avaliações complementares, pode auxiliar na identificação do impacto da frenectomia lingual sobre esses aspectos. A análise acústica da fala permite identificar as características sonoras específicas, como os formantes, que são eventos acústicos que podem ser sistematicamente associados à posição da língua durante a produção do som^(10,11)^.

Já a ultrassonografia (USG), por sua vez, possibilita a avaliação dos movimentos da língua por meio da colocação de um transdutor na região submandibular, gerando imagens que evidenciam a magnitude dos movimentos linguais durante a articulação dos sons e coordenação dos movimentos rápidos e síncrônicos da língua^(12,13)^.

O uso de instrumentos como a USG e Análise Acústica da fala em pacientes com anquiloglossia pode enriquecer a avaliação e o acompanhamento terapêutico nessa área. O presente relato de caso buscou uma análise detalhada da relação entre os aspectos anatomofuncionais da língua e essas avaliações, que possibilitam a compreensão sobre a mobilidade da língua durante a produção da fala. Além disso, esses instrumentos podem ser incorporados à rotina clínica do profissional tanto na avaliação como no acompanhamento terapêutico com biofeedback em pacientes com anquiloglossia.

Desse modo, o objetivo da pesquisa foi verificar o efeito da frenectomia lingual nos aspectos anatomofuncionais da língua, nas características fonético-acústicas e na magnitude de movimentação de língua nos fones [ɾ] e [l] pós frenectomia lingual.

## APRESENTAÇÃO DO CASO CLÍNICO

Esta pesquisa foi aprovada pelo Comitê de Ética e Pesquisa da instituição sob parecer nº 6.588.482. O Termo de Consentimento Livre e Esclarecido - TCLE foi apresentado e assinado pelos responsáveis legais da participante do estudo. O presente estudo de caso seguiu as recomendações do *guideline* internacional para publicações de relato de caso CARE^(14)^.

Participou deste estudo uma paciente do sexo feminino com 10 anos de idade com alteração do frênulo lingual. Foi aplicado o Protocolo para Avaliação de Frênulo Lingual^(15)^.

Na anamnese, a queixa principal referia-se à fala, especialmente à produção do fone tepe alveolar vozeado [ɾ]. Não havia antecedentes familiares com alteração de frênulo lingual, não possuía problemas de saúde, respiratórios, mastigatórios e deglutitórios. Não apresentava alterações vocais, auditivas ou fonológicas e não possuía histórico de frenectomia lingual prévia. Com relação a sucção, a genitora relatou uma amamentação sem desconfortos por mais de um ano, período em que se iniciou o desmame.

A seguir, serão descritas as avaliações realizadas com a paciente. Todas foram conduzidas em três momentos: antes da frenectomia lingual (T1), após 7 dias da frenectomia (T2) e após 14 dias da frenectomia (T3). O Quadro 1 apresenta, de forma esquemática, os tempos avaliativos deste estudo e os procedimentos realizados em cada período.

**Quadro 1:** Momentos avaliativos e procedimentos realizados

Pré-frenectomia lingual (T1)		Após 7 dias da frenectomia lingual (T2)	Após 14 dias da frenectomia lingual (T3)
Procedimentos realizados		Procedimentos realizados	Procedimentos realizados
Anamnese;	FRENECTOMIA LINGUAL	Avaliação anatomofuncional;	Avaliação anatomofuncional;
Avaliação anatomofuncional;			
		Avaliação acústica;	Avaliação acústica;
Avaliação acústica;			
Avaliação Ultrassonográfica;			
		Avaliação Ultrassonográfica;	Avaliação Ultrassonográfica;

### Avaliação anatomofuncional da língua

O exame clínico foi iniciado por meio da mensuração da abertura máxima de boca e abertura máxima de boca com o ápice da língua tocando na papila incisiva, utilizando um paquímetro digital da marca *Electronic Digital Caliper*.

Em seguida, foi realizado o exame observacional do frênulo lingual, a partir da elevação da língua. Foram observados forma da ponta de língua, fixação do frênulo lingual no assoalho da boca e na face inferior da língua, além do comprimento e espessura do frênulo lingual.

Além disso, foi realizada a avaliação funcional com base nas seguintes observações: mobilidade da língua; posição da língua em repouso e durante a fala; avaliação da fala considerando os aspectos de omissão, substituição e/ou distorção; abertura da boca durante a fala; participação dos lábios; movimento mandibular; velocidade e precisão da fala.

Na prova de fala, foram coletadas amostras de fala informal, fala automática e a nomeação de figuras, utilizando as pranchas do próprio protocolo.

### Avaliação acústica da fala

A coleta acústica da fala da criança foi realizada por meio do software PRAAT, na versão (6.4.05)^(16)^. As configurações do espectrograma do referido software foram ajustadas para geração de espectrogramas do tipo *wide band*. No menu *Spectrum*, selecionou-se *Spectrogram settings,* na opção *Wide-band,* o valor da *window length* foi alterado para 0.0043, o que possibilitou a extração dos formantes dos fones-alvo.

A paciente foi posicionada sentada em uma poltrona, com o tronco ereto e o olhar voltado para o horizonte. Um microfone do tipo Karsect HT-9 foi ajustado com distância de aproximadamente 3 centímetros da cavidade oral. O microfone estava conectado a um adaptador Andrea PureAudio USB, que, por sua vez, foi acoplado ao computador do avaliador, o qual operava o software PRAAT.

A paciente foi orientada a emitir duas frases-veículo: uma contendo o fones-alvo [ɾ] e outra com o fone [l]. As frases foram criadas especialmente para este estudo, com o objetivo de gerar amostras de fala inseridas em um contexto fonético semelhante, permitindo a extração dos formantes dos fones [ɾ] e [l] das sílabas tônicas dos vocábulos *parada* e *palada*.

As frases utilizadas foram: “diga parada baixinho” e “diga palada baixinho”. As emissões foram gravadas no software para posterior análise e extração de medidas formânticas, bem como do tempo de duração da emissão dos vocábulos.

Para a análise acústica no software PRAAT, as palavras “diga” e “baixinho” foram removidas de ambas as frases-veículo, uma vez que sua função era apenas fornecer um contexto fonético semelhante. A partir desses recortes iniciais, foi extraída a medida de duração dos vocábulos [parada] e [palada].

Posteriormente, foram extraídos das sílabas tônicas [ɾa] e [la] dos vocábulos, e, em seguida, as vogais foram removidas, permanecendo apenas os fone-alvo [ɾ] e [l]. Com esses segmentos isolados no espectrograma, o cursor foi posicionado no centro da emissão, e os formantes F1, F2 e F3 foram extraídos.

### Avaliação ultrassonográfica dos movimentos da língua

Na avaliação ultrassonográfica, foi utilizado o software *Articulate Assistant Advanced* (AAA), versão 217.02. O software estava instalado em um computador da marca *Dell* e acoplado a um transdutor de ultrassonografia. A paciente foi posicionada sentada em uma poltrona, com o tronco formando um ângulo de 90° e olhar voltado para o horizonte.

Para a captação dos movimentos da língua durante a produção dos fones [ɾ] e [l], foram utilizadas frases-veículo específicas. Optou-se por sentenças em que os fones-alvo estivessem inseridos entre sons de fácil análise na imagem ultrasssonográfica. Assim, escolheu-se a vogal [a], por apresentar os seguintes parâmetros articulatórios: é uma vogal baixa em relação à altura da língua na dimensão vertical, central quanto ao deslocamento anteroposterior da língua, aberta no que se refere ao movimento da mandíbula, não-arredondada em relação ao posicionamento dos lábios e oral quanto ao movimento de fechamento do véu palatino^(4)^.

Os comandos foram “Repita: diga ara, diga ara, diga ara” e “Repita: diga ala, diga ala, diga ala”. As imagens ultrassonográficas foram gravadas para posterior extração das medidas da magnitude do movimento de língua nas porções anterior, média e posterior, durante a produção dos fones. Essas medidas foram obtidas por meio do traçado da superfície da língua, utilizando a ferramenta denominada *spline*.

Nas análises ultrassonográficas da língua, foram coletados os valores das distâncias entre o ponto base do transdutor e as porções anterior, média e posterior da língua, tanto na posição mais baixa (inicio da articulação do fone-alvo) quanto na posição mais elevada (articulação completa do fone-alvo). Esse procedimento foi realizado para ambos os fones, [ɾ] e [l], nos três momentos avaliativos: T1, T2 e T3 (Figura 1).

**Figura 1:** Imagem ultrassonográfica com exemplo da extração das medidas anterior, média e posterior da língua

**Legenda:** PA = Porção Anterior da Língua; PM = Porção Média da Língua; PP = Porção Posterior da Língua; SL = Superfície da Língua; MA = Medida anterior da língua; MM = Medida média da língua; MP = Medida posterior da língua; PB = Ponto Base. Fonte: dos autores, 2024

### Frenectomia lingual

Após a realização de todas as avaliações, a paciente foi encaminhada para a frenectomia lingual, a qual foi realizada por um cirurgião-dentista com experiência na área e integrante do grupo de pesquisa. A paciente retornou para reavaliação após 7 dias (T2) e 14 dias (T3) de cicatrização.

Foram realizadas a documentação fotográfica fonoaudiológica, a reavaliação anatomofuncional da língua, a avaliação acústica e avaliação ultrassonográfica, com o objetivo de permitir comparações e análises posteriores.

## RESULTADOS

### Avaliação anatomofuncional da língua

Em T1, no exame clínico do Protocolo para Avaliação do Frênulo Lingual, a paciente obteve 5 pontos, sendo que o melhor resultado seria 0 e o pior 8, sendo 3 pontos o valor de referência para alteração de frênulo lingual. Durante a elevação da língua, a ponta apresentava formato retangular e a fixação do frênulo no assoalho da boca localizava-se na crista alveolar inferior. Já a fixação do frênulo lingual na face inferior da língua estava entre o terço médio e o ápice. Quanto à espessura, o frênulo lingual foi classificado como delgado.

Após a frenectomia lingual, em T2 e T3, observou-se aumento na elevação da língua na cavidade oral, com modificação do seu formato, sendo essas mudanças mais evidentes em T3 (Figura 2).

**Figura 2:** Elevação da língua antes e após a frenectomia lingual

**Legenda:** A = elevação da língua antes da frenectomia lingual; B = elevação da língua após 7 dias da frenectomia lingual; C = elevação da língua após 14 dias da frenectomia lingual. Fonte: dos autores, 2024

Na avaliação da abertura de boca com o paquímetro, verificou-se aumento progressivo das medidas em milímetros (mm) entre os tempos avaliativos. Em T1, a abertura máxima de boca com ápice da língua tocando na papila incisiva foi de 9 mm; em T2, de 9.3 mm; e, em T3 de 10 mm. A relação entre a abertura máxima de boca e abertura máxima de boca com o ápice da língua tocando na papila incisiva foi de 27,2% em T1, aumentando para 30,3% em T3.

No tempo avaliativo T1, nas provas funcionais, a paciente obteve 24 pontos, em uma escala cujo melhor resultado é 0 e o pior, 41, sendo 20 pontos o valor indicativo de possível interferência do frênulo lingual na movimentação da língua. Na prova de mobilidade, a paciente executou de forma aproximada ou não executou as tarefas: sugar a língua no palato, vibrar o ápice da língua, protrair, tocar o lábio superior com o ápice da língua e tocar as comissuras com a ponta da língua.

Durante a protrusão, a ponta da língua apresentava um padrão de curvatura para baixo. Ao tocar o lábio superior com o ápice, observou-se fechamento labial acentuado. Ao tentar tocar as comissuras labiais, notou-se assimetria entre os lados e tendência da ponta da língua a virar para baixo. Durante o repouso, não foi possível observar a posição da língua, uma vez que a paciente mantinha um bom vedamento labial.

As avaliações funcionais citadas acima foram repetidas nos momentos T2 e T3. Observou-se a permanência dos padrões de execução aproximada ou não execução presentes no pré-operatório. No entanto, a paciente demonstrou maior amplitude e destreza na execução do comando de toque nas comissuras labiais (Figura 3).

**Figura 3:** Toque da comissura labial direita com a ponta da língua

**Legenda:** A = Toque da comissura labial direita com a ponta da língua antes da frenectomia lingual; B = Toque da comissura labial direita com a ponta da língua após 7 dias da frenectomia lingual; C = Toque da comissura labial direita com a ponta da língua após 14 dias da frenectomia lingual. Fonte: dos autores, 2024

Outros aspectos observados na fala da paciente em T1 incluíram abertura de boca reduzida, posição da língua no assoalho tanto em repouso como durante a fala e precisão articulatória comprometida. Nas reavaliações (T2 e T3), observaram-se modificações na dinâmica mandibular durante a fala, mais evidentes em T3.

Na prova de fala, em T1, a paciente obteve 9 pontos, sendo 0 o melhor resultado e 12 o pior. Foi observada distorção do fone [ɾ] em todas as amostras de fala. No fone [l], não se observou distorção fonética, indicando boa articulação desse som. Em T3, apesar da frenectomia lingual, manteve-se a distorção fonética do fone [ɾ].

### Avaliação acústica da fala

Na avaliação acústica, foi identificado um aumento na duração da emissão dos vocábulos-veículo quando comparadas a avaliação inicial e as reavaliações. A Tabela 1 apresenta os valores, em milissegundos (ms), referentes à duração dos vocábulos antes e após a frenectomia lingual.

**Tabela 1:** Duração da emissão dos vocábulos veículos [parada] e [palada] antes e após a frenectomia lingual

DURAÇÃO DO VOCÁBULO ALVO	[PARADA]	[PALADA]
T1	0,42 segundos	0,37 segundos
T2	0,43 segundos	0,57 segundos
T3	0,69 segundos	0,63 segundos

Legenda: T1 = avaliação antes da frenectomia; T2 = avaliação após 7 dias da frenectomia; T3 = avaliação após 14 dias da frenectomia.

Fonte: dos autores, 2024

A Tabela 2 apresenta os valores, em hertz (Hz), dos formantes para os fones [ɾ] e [l] antes e após a frenectomia lingual.

**Tabela 2:** Medidas formânticas antes e após a frenectomia lingual

FORMANTES	Fone [ɾ] em Hz			Fone [l] em Hz		
	T1	T2	T3	T1	T2	T3
F1	791,6	754,6	807,6	552,9	507,8	587,1
F2	2032,1	2198,9	2324,8	958,1	1386,2	1045,2
F3	3003,7	3030,5	2965,2	2219,7	1535,6	1296,8

**Legenda:** T1 = avaliação antes da frenectomia; T2 = avaliação após 7 dias da frenectomia; T3 = avaliação após 14 dias da frenectomia. Fonte: dos autores, 2024

Em T2 e T3, observaram-se modificações nas medidas formânticas. Os valores de F1 e F2 aumentaram em T3 para ambos os fones. Além disso, verificou-se variabilidade nas medidas formânticas entre T2 e T3. Em relação ao formante F3, tanto para [ɾ] quanto para [l], observou-se redução nos valores, sendo essa diminuição mais acentuada para o fone [l].

Nas medidas formânticas referentes à transição dos fones-alvo para a vogal [a], também foram observadas alterações. Houve aumento nos valores de F1, F2 e F3 para ambos os fones, quando comparada a avaliação inicial com os momentos pós-frenectomia lingual.

### Avaliação ultrassonográfica dos movimentos de língua na fala

Foram observadas modificações nas medidas extraídas das porções anterior, média e posterior da língua durante os momentos avaliativos, T1, T2 e T3. Para o fone [ɾ], houve aumento das medidas em todas as porções da língua em todos os momentos avaliativos (Tabela 3).

**Tabela 3:** Medidas ultrassonográficas da língua em sua posição baixa e alta durante a emissão dos fones-alvo

	LÍNGUA BAIXA					
	[ɾ]			[l]		
	T1	T2	T3	T1	T2	T3
Porção anterior	1,35	1,39	1,60^*^	1,58	1,64	1,63*
Porção média	1,89	1,91	1,98*	1,91	2,04	1,64
Porção posterior	1,58	1,99	1,70*	1,78	2,40	1,75*
	LÍNGUA ALTA					
	[ɾ]			[l]		
	T1	T2	T3	T1	T2	T3
Porção anterior	1,72	1,70	1,78*	1,98	1,81	1,90
Porção média	1,75	2,19	2,18*	1,96	2,36	2,04
Porção posterior	1,11	2,92	1,70*	1,92	2,35	1,48

**Legenda:** T1 = avaliação antes da frenectomia; T2 = avaliação após 7 dias da frenectomia; T3 = avaliação após 14 dias da frenectomia

* Medidas ultrassonográficas que aumentaram após as reavaliações

Fonte: dos autores, 2024

Para o fone [l], observou-se assistematicidade nas medidas. Na comparação entre T1 e T2, verificou-se aumento na elevação das porções anterior, média e posterior da língua na posição da língua baixa, bem como aumento na elevação das porções média e posterior na posição da língua alta. Esses achados indicam uma postura de língua mais elevada em T2, a qual, contudo, não se manteve em todas as regiões em T3 (Tabela 3).

Além disso, em T3, verificou-se redução da diferença entre as posições de língua baixa e alta para a produção do fone [ɾ] nas porções anterior e posterior da língua. Para o fone [l], essa redução foi observada apenas na porção anterior da língua (Tabela 3).

## DISCUSSÃO

Neste relato de caso, foi descrito o acompanhamento de uma paciente de 10 anos com alteração no frênulo lingual, submetida à frenectomia lingual, à avaliação acústica da fala e avaliação ultrassonográfica dos movimentos da língua durante a fala.

As modificações clínicas observadas, como maior elevação da língua dentro da cavidade oral, mudança no formato da ponta da língua, aumento da abertura de boca com o ápice da língua tocando na papila incisiva e melhor desempenho no toque do ápice da língua nas comissuras labiais, corroboram com achados anteriores que observaram melhora da movimentação tridimensional da língua na cavidade oral após sete dias da frenectomia lingual^(17)^. Da mesma forma, foi verificado que, quando há uma frenectomia lingual bem-sucedida, melhorias na movimentação da língua do paciente são evidenciadas de forma imediata^(18)^.

Na análise acústica da fala, o aumento no tempo da pronúncia dos vocábulos *parada* e *palada* pode ser atribuído à provável maior amplitude de movimento da língua e maior precisão quanto ao ponto articulatório. Este achado está em concordância com a literatura, visto que a anquiloglossia tende a restringir a movimentação da língua, levando à omissão ou distorção dos sons da fala e resultando em uma articulação imprecisa^(3)^.

Quanto à avaliação dos formantes dos fones [ɾ] e [l], o aumento nos valores de F1 e F2, entre os momentos T1 e T3, pode estar associado à maior abertura mandibular e verticalização da língua, bem como à maior anteriorização lingual. Esses achados estão de acordo com a literatura, que aponta que quanto maior o valor de F1, maior o deslocamento da mandíbula no sentido vertical e quanto maior o valor de F2, mais anteriorizada está a língua. Já no formante F3, a diminuição do seu valor está associada ao aumento do tamanho das cavidades estabelecidas pela posição da língua^(10)^. Sendo assim, entende-se que a frenectomia lingual acarretou modificações positivas na posição da língua, detectadas acusticamente.

Neste estudo, foi observado que a análise acústica da duração das palavras desempenhou um papel relevante na detecção de mudanças na articulação dos fones durante a fala. A análise dos formantes apresentou resultados consistentes para os formantes F1 e F2, indicando ser uma ferramenta útil para auxiliar na avaliação e acompanhamento terapêutico da fala do paciente.

A análise ultrassonográfica da língua identificou maior elevação durante a produção do fone [ɾ], indicando um impacto positivo da frenectomia lingual na amplitude dos movimentos linguais na fala, corroborando com os achados acústicos. Quanto ao fone [l], a inconsistência nos resultados pode ser atribuída à tendência de maior elevação da língua após o procedimento cirúrgico; porém, esse padrão não se manteve, visto que esse fone não apresentou alterações significativas na fala, o que levou a um aumento imediato da amplitude, seguindo de retorno ao padrão habitual de produção.

Ao comparar as medidas da posição da língua baixa e elevada, a redução na diferença entre essas medidas indica uma posição mais elevada da língua durante a produção dos fones [ɾ] e [l]. No que diz respeito à articulação do [ɾ], houve um aumento nas medidas da porção anterior e posterior da língua. Esse aumento sugere uma elevação do ápice da língua durante a fala, o que pode favorecer a reabilitação desse fone. Em relação à porção posterior da língua, é possível que este achado indique possíveis compensações articulatórias, mais evidentes em indivíduos com anquiloglossia, como encontrado em estudos anteriores^(19)^.

É importante ressaltar que, apesar dos ganhos imediatos observados após a frenectomia lingual, a distorção na articulação do fone [ɾ] persistiu. Assim, torna-se fundamental o acompanhamento fonoaudiológico para promover o equilíbrio miofuncional, facilitando a adequação da fala da paciente. Recomenda-se que esse acompanhamento se inicie após a cicatrização do procedimento, que ocorre em aproximadamente quatorze dias, pois sabe-se que a liberação anatômica, por si só, não assegura a reabilitação funcional, e novas compensações podem surgir na ausência de intervenção fonoaudiológica^(19)^.

A principal limitação deste estudo reside na análise acústica dos formantes e sua associação com a movimentação da língua e mandíbula durante a produção da fala. Embora a associação acústica com a produção de vogais esteja bem estabelecida na literatura, essa relação ainda não está claramente definida para os sons líquidos do português brasileiro. No entanto, apesar da ausência dessas evidências consolidadas, neste relato de caso observou-se que os achados acústicos dos formantes indicaram maior movimentação da língua nos eixos vertical e anteroposterior após a frenectomia lingual. Além disso, a USG possibilitou a identificação dessas movimentações por meio de imagens e medidas, corroborando a avaliação clínica fonoaudiológica. Dessa forma, a análise acústica e USG mostram-se instrumentos relevantes para a prática clínica do fonoaudiólogo.

## COMENTÁRIOS FINAIS

Neste relato de caso, referente a uma criança com 10 anos, diagnosticada com anquiloglossia e submetida à frenectomia lingual, foi possível observar que a avaliação acústica da fala e a avaliação ultrassonográfica da língua são instrumentos que podem ser incorporados à prática clínica fonoaudiológica para complementar a avaliação clínica e o processo terapêutico. Esses instrumentos, ao fornecerem medidas objetivas relacionadas à movimentação da língua na cavidade oral, demonstram potencial para acompanhar a evolução clínica do paciente, além de servirem como recurso de biofeedback durante a intervenção fonoaudiológica.
